# Enhanced Methane Sensing Properties of WO_3_ Nanosheets with Dominant Exposed (200) Facet via Loading of SnO_2_ Nanoparticles

**DOI:** 10.3390/nano9030351

**Published:** 2019-03-04

**Authors:** Dongping Xue, Junjun Wang, Yan Wang, Guang Sun, Jianliang Cao, Hari Bala, Zhanying Zhang

**Affiliations:** 1 The Collaboration Innovation Center of Coal Safety Production of Henan Province, Henan Polytechnic University, Jiaozuo 454000, China; xdongping1231@126.com (D.X.); yanwang@hpu.edu.cn (Y.W.); mcsunguang@163.com (G.S.); caojianliang@hpu.edu.cn (J.C.); hari@hpu.edu.cn (H.B.); 2 Institute of Materials Science and Engineering, Henan Polytechnic University, Jiaozuo 454000, China; 3 Institute of Physics & Electronic Information Engineering, Henan Polytechnic University, Jiaozuo 454000, China; 4 Institute of Safety Science and Engineering, Henan Polytechnic University, Jiaozuo 454000, China

**Keywords:** SnO_2_-loaded WO_3_ nanosheets, methane sensing, heterojunction, exposed (200) facet

## Abstract

Methane detection is extremely difficult, especially at low temperatures, due to its high chemical stability. Here, WO_3_ nanosheets loaded with SnO_2_ nanoparticles with a particle size of about 2 nm were prepared by simple impregnation and subsequent calcination using SnO_2_ and WO_3_·H_2_O as precursors. The response of SnO_2_-loaded WO_3_ nanosheet composites to methane is about 1.4 times higher than that of pure WO_3_ at the low optimum operating temperature (90 °C). Satisfying repeatability and long-term stability are ensured. The dominant exposed (200) crystal plane of WO_3_ nanosheets has a good balance between easy oxygen chemisorption and high reactivity at the dangling bonds of W atoms, beneficial for gas-sensing properties. Moreover, the formation of a n–n type heterojunction at the SnO_2_-WO_3_ interface and additionally the increase of specific surface area and defect density via SnO_2_ loading enhance the response further. Therefore, the SnO_2_-WO_3_ composite is promising for the development of sensor devices to methane.

## 1. Introduction

The gas detection of methane is significantly important in coalmine production, usage of natural gas, atmospheric monitoring, etc. However, due to the high chemical stability of the C–H bond in methane, methane detection is extremely difficult, especially at low temperatures. Therefore, it is important to develop a reliable, sensitive sensor that can detect methane at low temperatures. Metal oxide semiconductor (MOS) sensors have attracted extensive attention due to their low cost, high sensitivity, fast response/recovery, and easy integration [1,2,3,4]. However, the applications of these MOS sensors are limited due to their high operating temperature and poor stability [5,6,7]. In order to reduce the operating temperature and improve the stability and sensitivity of different MOS gas sensors, methods such as noble metal/transition metal doping, heterojunction formation and unique surface morphology have been studied [8,9,10,11,12,13].

WO_3_ is one of the most promising gas-sensitive materials due to its moderate energy band, rich oxygen vacancies and high response. In particular, since Akiyama et al. started their work, the gas-sensing characteristics of WO_3_ have been well studied due to the high sensitivity of WO_3_ to various gases [14]. It is well known that the gas-sensing properties of MOS materials are considerably dependent on their morphology [15,16]. Hence, many unique WO_3_ nanostructures, such as nanosheets [17,18,19], nanorods [20], nanowires [21,22] and nanospheres [23,24], have been synthesized to improve the gas sensitivity of gas sensors. Among these topographies, 2D nanosheets having a flat surface and a regular shape have attracted great attention in various fields due to their good optical and electrical properties [25]. SnO_2_ has become one of the most widely studied nanomaterials due to its unique properties [2,26]. The SnO_2_-WO_3_ hybrid structure has received great attention because SnO_2_ and WO_3_ have different degrees of reaction to various redox gases, moderate resistivity, significant catalytic activity, high stability, low cost and unique gas-sensing characteristics [27,28,29,30,31,32,33,34]. When an n–n type heterojunction is formed at the interface between SnO_2_ and WO_3_, the Fermi levels of the two constitute materials across the heterojunction equilibrate to the same energy level leading to charge transfer and consequently the formation of a space charge region serving as the basis of the increase of sensor response [35]. However, very few studies have been reported regarding methane sensing employing the SnO_2_-WO_3_ hybrid structure.

In this work, we investigated the methane gas-sensing properties of SnO_2_-WO_3_ hybrid structures. We successfully prepared WO_3_ nanosheets using a simple one-step hydrothermal method, and added a small number of SnO_2_ nanoparticles with a particle size of about 2 nm using the impregnation method to obtain SnO_2_-WO_3_ nanocomposites. Our study showed that the SnO_2_-WO_3_ nanocomposite had higher sensitivity to methane than pure WO_3_ nanosheets and that the optimum operating temperature of both sensors was relatively low at 90 °C. The crystal structure, morphology, specific surface area, and pore-size distribution of the as-prepared pure and SnO_2_-loaded WO_3_ nanosheets were investigated using various characterization tools. Gas-sensing properties were tested systematically and the gas-sensing mechanism was thoroughly discussed with the focus on the influence of the heterojunction and the observed dominant surface facet of the WO_3_ nanosheets.

## 2. Experimental Details

### 2.1. Preparation of WO_3_ H_2_O Nanosheets, SnO_2_ Nanoparticles and Their Composite

All chemical reagents used in the experiments, including sodium tungstate (Na_2_WO_4_·2H_2_O, 99.5%), stannic chloride pentahydrate (SnCl_4_·5H_2_O, 99%), nitric acid (HNO_3_, 65%), polyethylene glycol 400 (PEG-400), sodium hydroxide (NaOH, 98%), aqueous ammonia (NH_3_·H_2_O 25–28%), and absolute ethanol were of analytical grade and as received without any further purification. Distilled water was used throughout the experiments.

Both the WO_3_·H_2_O nanosheets and the SnO_2_ nanoparticles were synthesized through a one-step hydrothermal method. In a typical synthesis of WO_3_·H_2_O nanosheets, 0.323 g sodium tungstate (Na_2_WO_4_·2H_2_O) was dissolved in 15 mL of distilled water under continuous stirring, and 10 mL of HNO_3_ solution was added. After vigorous stirring for 10 min, the obtained mixture was transferred into a 50 mL stainless autoclave lined with a Teflon vessel and maintained at 180 °C for 12 h. After naturally cooling down, the precipitates were collected by centrifugation, washed with distilled water and absolute ethanol for several times and then dried in the air at 60 °C for 12 h.

For synthesis of SnO_2_ nanoparticles, 5 mL of SnCl_4_·5H_2_O ethanol solution (1 M) was added to 90 mL of 50% diluted ethanol solution and 5 mL of NH_3_·H_2_O was added to form a homogeneous suspension under magnetic stirring. The suspension was transferred into a 100 mL stainless autoclave lined with a Teflon vessel and maintained at 150 °C for 24 h. After naturally cooling down, the as-synthesized sample was collected, washed, and dried as described for WO_3_·H_2_O.

To prepare the SnO_2_-WO_3_ composites, the above synthesized WO_3_·H_2_O nanosheets 0.04 g, SnO_2_ nanoparticles 0.002 g were dissolved in 5 mL of distilled water, underwent ultrasonic treatment for 2 h and then were dried at 60 °C for 6 h. Finally, the obtained precipitates were annealed at 450 °C for 2 h to strengthen the chemical bonding between SnO_2_ and WO_3_.

### 2.2. Material Characterization

The crystalline structure and phase of the samples were investigated by X-ray diffraction (XRD, Bruker-AXS D8, Bruker, Madison, WI, USA) with Cu Kα radiation at 40 kV and 150 mA in a scanning range of 20–80° (2*θ*) in steps of 0.02°. The morphologies and nanostructures were investigated by field-emission scanning electron microscopy (FESEM, Quanta™ FEG 250) (FEI, Eindhoven, The Netherlands) and transmission electron microscopy (TEM) analysis is performed on a Tecnai G2 F20 microscope (FEI, Eindhoven, The Netherlands) operating at 200 kV. UV-vis absorption spectra were obtained on a UV-vis diffuse reflection spectrometer (TU1901) (General Analytical Instruments Company, Beijing, China). N_2_ adsorption–desorption was performed on a Quantachrome Autosorb-iQ sorption analyzer (Quantachrome, Boynton Beach, FL, USA). The specific surface area of the products was calculated following the multi-point Brunauer–Emmett–Teller (BET) procedure.

### 2.3. Gas-Sensing Measurement

**Figure 1:**
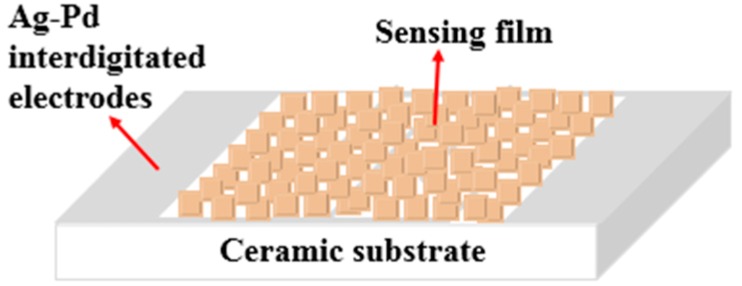
Schematic diagram of a gas sensor.

The fabrication of the sensor is similar to our previously reported work [36,37]. The samples were mixed with distilled water to form a homogeneous paste and coated onto a ceramic substrate (13.4 mm × 7 mm) with an Ag-Pd interdigitated electrode (Figure 1). Gas-sensing tests were carried out on an intelligent gas-sensing analysis system of CGS-4TPS (Beijing Elite Tech Co., Ltd., Beijing, China) under laboratory conditions (20 RH%, 25 °C). The sensor response was defined as *R*_a_/*R*_g_, where *R*_a_ and *R*_g_ represent the resistance of the sensor in air and target gas, respectively.

## 3. Results and Discussion

### 3.1. Characterizations of the As-Prepared Samples

**Figure 2:**
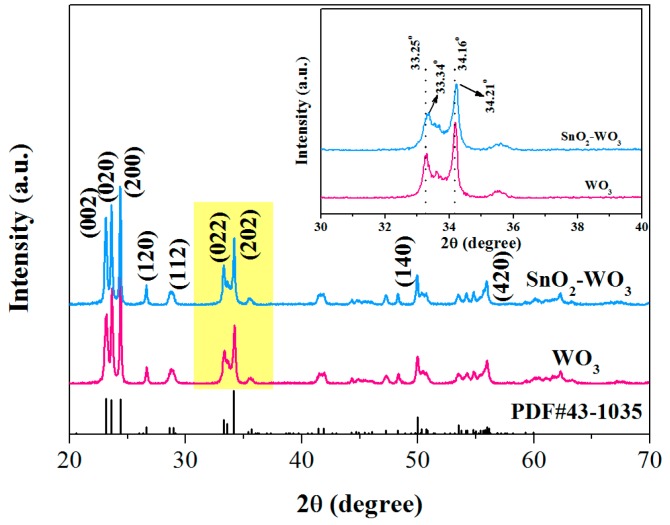
XRD patterns of the as-prepared pure WO_3_ and SnO_2_-WO_3_ nanocomposite.

To investigate the effect of the loading of SnO_2_ nanoparticles on WO_3_, XRD analysis of the samples is shown in Figure 2. All the diffraction peaks of pure WO_3_ were in complete agreement with monoclinic WO_3_ (JCPDS file No. 43−1035), and no diffraction peaks of other impurities was found, indicating that the pure sample was a crystalline phase with high-purity WO_3_. No SnO_2_ diffraction peak was found in the XRD diffraction pattern of SnO_2_-WO_3_ sample, presumably due to its low load (5 wt.%). However, the peak position was shifted compared to the pure WO_3_ diffraction peak, as shown in the inset of Figure 2; the shift in peak positions Δ(2*θ*) was found to be ~0.09° and 0.05° for SnO_2_-WO_3_ with respect to pure WO_3_, speculatively due to the interaction between SnO_2_ and WO_3_.

**Figure 3:**
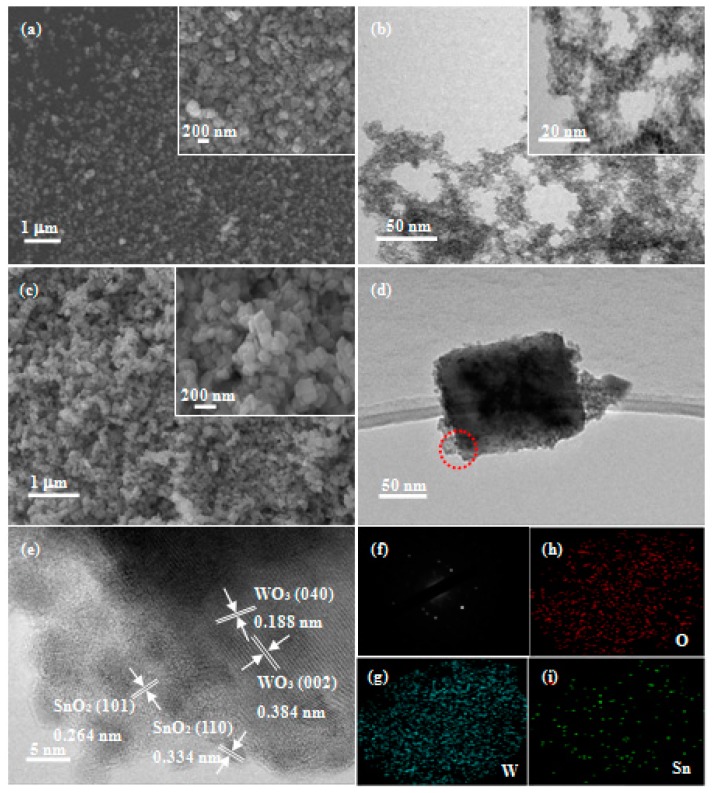
Field-emission scanning electron microscopy (FESEM) images of (**a**) WO_3_ nanosheets and (**c**) SnO_2_-WO_3_ nanosheets. FETEM images of (**b**) SnO_2_ nanoparticles and (**d**) SnO_2_-WO_3_ nanosheets. (**e**) High-resolution transmission electron microscopy (HRTEM) image, (**f**) selected area electron diffraction (SAED) image and (**g**–**i**) energy-dispersive X-ray spectroscopy (EDS) image of SnO_2_-WO_3_ nanosheets.

The morphology and structural characteristics of the samples were characterized by FESEM and TEM as shown in Figure 3. Figure 3a,c shows the typical FESEM images of WO_3_ nanosheets and SnO_2_-WO_3_ nanocomposites, and the insets are the respective enlarged FESEM images. It can be clearly seen that both samples were composed of nanosheets with a side length of about 100 nm. The SnO_2_ nanoparticles in the composite were too small and too few to be seen with limited magnification. The pure WO_3_ nanosheets had a more uniform distribution, more uniform particle size, and more regular and smoother surface than the SnO_2_-loaded ones. Figure 3b shows the TEM image of the SnO_2_ nanoparticles, the inset of which is the TEM image of the same sample with an even higher magnification. The SnO_2_ nanoparticles have a particle size of about 2 nm and are evenly distributed. Figure 3d presents the TEM image of the SnO_2_-WO_3_ nanocomposite. As can be seen, the SnO_2_ nanoparticles are preferably dispersed on the WO_3_ nanosheets. Figure 3e shows the high-resolution transmission electron microscopy (HRTEM) image of the SnO_2_-WO_3_ nanocomposite, where the lattice spacing of 0.188 nm and 0.384 nm can be indexed to the (040) and (002) crystal planes of the monoclinic WO_3_ phase, respectively. Therefore, the dominant exposed facet of the WO_3_ nanosheets can be determined to be the (200) facet. The strongest diffraction peak in the XRD patterns is indexed to the (200) crystal plane indicating the preferential growth crystal plane, being consistent with the exposed surface facet. Moreover, the lattice spacing of SnO_2_ as 0.264 nm and 0.334 nm are indexed to the (101) and (110) crystal planes of the tetragonal rutile SnO_2_ phase, respectively. Figure 3f presents the selected area electron diffraction (SAED) diagram of a single SnO_2_-loaded WO_3_ nanosheet from Figure 3d. It can be clearly seen that the WO_3_ nanosheet is a single crystal, but the diffraction spots are not periodically distributed, probably due to the existence of two phases; firstly, WO_3,_ and secondly, SnO_2_. To further prove that the SnO_2_ is indeed loaded onto the WO_3_ nanosheets, the SnO_2_-WO_3_ nanocomposites were characterized by energy-dispersive X-ray spectroscopy (EDS) techniques as shown in Figure 3g–i, and it is obvious that W, O and Sn elements are present in the composite.

**Figure 4:**
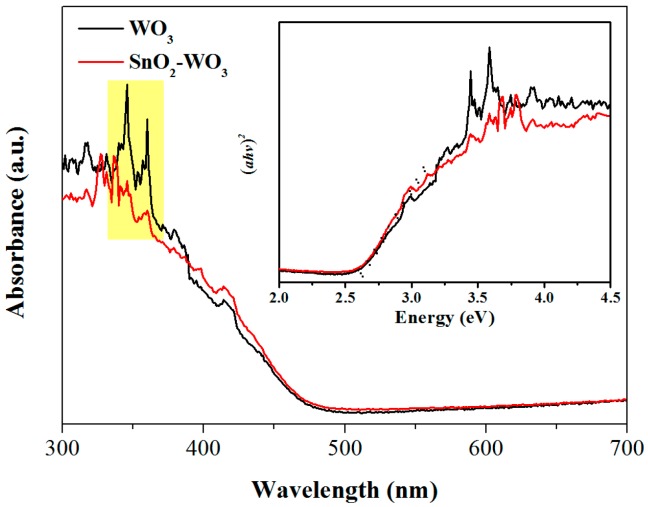
UV–vis absorption spectra of the pure WO_3_ and SnO_2_-WO_3_ nanosheets. The inset shows the relationship between (*αhv*)^2^ and *hv*.

UV-vis absorption spectra of pure and SnO_2_-loaded WO_3_ are shown in Figure 4. A red shift is observed for the absorption spectrum of SnO_2_-WO_3_ as compared to that of WO_3_. The inset of Figure 4 shows the relationship between (*αhv*)^2^ and photon energy *hv*, where α is the absorption coefficient, *h* the Planck constant, and *v* the light frequency. Extrapolating the part of the spectra near the absorption edge, the intersection with the abscissas is obtained as the band gap, 2.62 eV and 2.57 eV for pure and SnO_2_-loaded WO_3_, respectively. This indicates that the band gap of WO_3_ is narrowed due to the modification of SnO_2_ nanoparticles, beneficial for electron transition and therefore for the oxygen chemisorption at the surface. Meanwhile, the modified band structure of WO_3_ is proof of the heterojunction between SnO_2_ and WO_3_ [38,39,40]. Simultaneously, the shape change of the absorption spectrum for SnO_2_-WO_3_ composite at some wavelengths, e.g., 340–360 nm, indicates that the band structure of WO_3_ in the composite is modified by loading SnO_2_ and consequently, SnO_2_-WO_3_ composite is definitely not two individual materials without chemical bonding between them.

**Figure 5:**
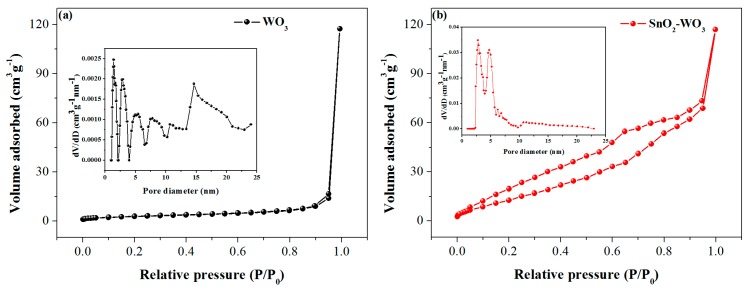
N_2_ adsorption and desorption isotherms of (**a**) pure WO_3_ nanosheets and (**b**) SnO_2_-WO_3_ nanosheets with their corresponding pore-size distribution (inset).

The specific surface area and pore-size distribution of the pure and SnO_2_-loaded WO_3_ were estimated by N_2_ adsorption–desorption. As shown in Figure 5a,b, two samples exhibit a IV-type adsorption isotherm with a H3-type hysteresis loop. The specific surface areas of pure WO_3_ and SnO_2_-WO_3_ were calculated by the BET method to be 10.5 m^2^/g and 57.7 m^2^/g, respectively. From the inset of Figure 5a, the pore-size distribution range of pure WO_3_ is estimated to be mainly 1.3 nm–14.6 nm. According to the SEM image (Figure 3), these pores can be attributed to random stacking of WO_3_ nanosheets. The inset of Figure 5b reveals the main pore size of the SnO_2_-WO_3_ sample ranges from 2.6 nm to 4.7 nm. It can be inferred from the TEM image that these pores are mainly formed by the distribution of SnO_2_ nanoparticles on WO_3_ nanosheets, and the loading of the SnO_2_ nanoparticles makes the pore-size distribution of the composite more uniform.

### 3.2. Gas-Sensing Properties

**Figure 6:**
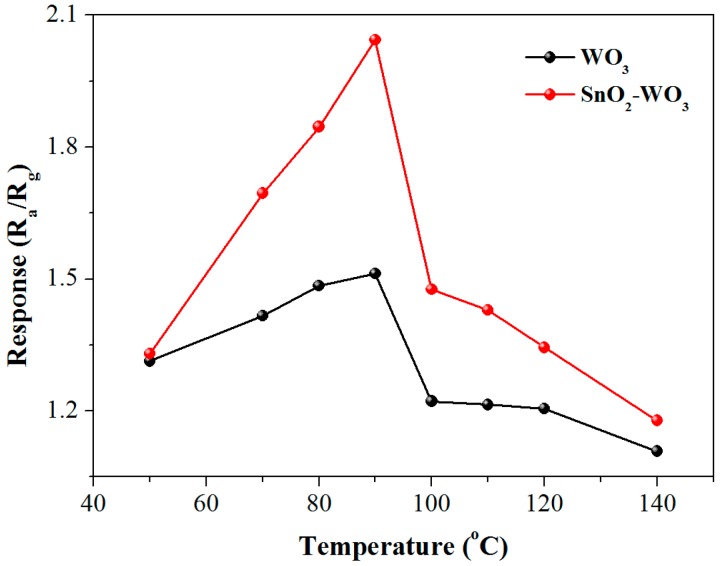
Response of pure WO_3_ and SnO_2_-WO_3_ sensors to 500 ppm methane at different operating temperatures.

**Figure 7:**
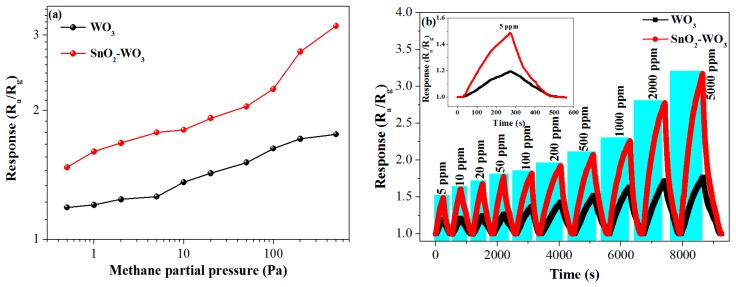
(**a**) Response curve at different methane partial pressures at 90 °C, (**b**) response/recovery curve of pure WO_3_ and SnO_2_-WO_3_ sensors at different methane concentrations at 90 °C.

**Figure 8:**
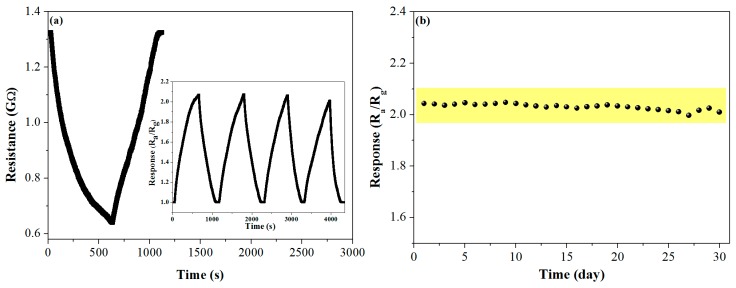
(**a**) Repeatability and (**b**) long-term stability of pure WO_3_ and SnO_2_-WO_3_ sensors towards 500 ppm methane at 90 °C.

Gas sensors based on pure and SnO_2_-loaded WO_3_ nanosheets were prepared and their series of gas-sensing properties for methane were investigated. Since working temperature has a great influence on the gas-sensing performance, the dependence of the sensor response on working temperature was investigated with 500 ppm of the methane concentration. The response firstly increased with an increasing operating temperature, up to an optimum operating temperature of 90 °C, then saturated and dropped with a further rising temperature (Figure 6). This may be because the chemisorbed oxygen reached the energy required to react with the methane molecules, so an effective reaction at the surface of the material caused a significant change in electrical resistance [41]. Importantly, the response of the SnO_2_-loaded WO_3_ sensor was enhanced as compared to that of pure WO_3_ in the temperature range of 50–140 °C, by a maximum factor of 1.4 at 90 °C, indicating that the loading of the SnO_2_ nanoparticles had an obvious effect of improving the gas sensitivity of the WO_3_-based methane sensor. The response dependent on the methane partial pressure at 90 °C was investigated for both sensors (Figure 7a) [42,43]. As expected, the response of both sensors increased with an increasing methane partial pressure. Both the rising slope and the response value of SnO_2_-loaded WO_3_ were greater than those of pure WO_3_. The dynamic response curves of the pure and SnO_2_-loaded WO_3_ sensor to different methane concentrations were measured (Figure 7b). The response of the SnO_2_-WO_3_ sensor was always higher than that of the pure WO_3_ sensor with increasing methane concentration and recovered to the base value when the sensor was exposed to air after multiple operation cycles. The inset in Figure 7b shows the amplified response/recovery curve for the methane concentration of 5 ppm. According to the inset, full recovery was achieved and the slope of the response decreases with time indicating the response was approaching saturation. The slow response/recovery was caused by the low working temperature and the strong stability of methane molecules. This phenomenon has been observed by other reports on the detection of methane [4,44,45]. Methods of achieving rapid response/recovery at low working temperatures still need to be investigated. From Figure 7b, it is clear that the initial slope of the dynamic response curve of the SnO_2_-WO_3_ sensor is larger than that of the pure WO_3_ sensor, presumably due to the increased number of active sites [46], which will be discussed in Section 3.3. Repeatability and long-term stability are also important parameters for the practical application of a sensor. As shown in Figure 8a,b, the SnO_2_-WO_3_ sensor could maintain response/recovery performance without major changes after four operation cycles, and the response was kept within 90% of the initial value during 30 days, revealing that the sensor had good repeatability and long-term stability.

### 3.3. Gas-Sensing Mechanism

The optimum operating temperature for methane detection in this work was relatively low at 90 °C. The stable adsorbed oxygen species on metal oxides was [O_2_^−^] at low temperatures <200 °C [47]. The basic principle of the gas-sensing mechanism was well described by the receptor function. In air, the atmospheric oxygen was adsorbed on the surface and ionized to be [O_2_^−^], trapping the electrons from the conduction band of the metal oxides leading to the formation of electron depletion region at the metal oxide surface. If the metal oxide was a n-type semiconductor, its resistance increased. When the target gas methane was introduced, it reacted with the adsorbed [O_2_^−^] as follows:

$${\mathrm{CH}}_{4}\left(\mathrm{gas}\right)+2\left[{O_{2}}^{-}\right]\left(\mathrm{adsorbed}\right)\to {\mathrm{CO}}_{2}+2H_{2}O+2e^{-}$$

releasing the electrons back into the metal oxides. Therefore, the resistance reduced. The response of the metal oxides was based on the resistance change via the oxygen adsorption and desorption.

A specific exposed facet of the sensing material should have specific physical and chemical properties and therefore offer characteristic sensing performance [48,49,50]. The dominant exposed facet of the WO_3_ nanosheets in this work was determined to be the (200) crystal plane as discussed above. Many investigations have been done regarding the comparison among the exposed facets (200), (020) and (002) in WO_3_ [51,52,53]. The (002) crystal plane of γ-WO_3_ is an oxygen-terminated facet, which contains exclusively unsaturated coordinated oxygen atoms. The chemisorption of the atmospheric oxygen is beneficial on such a facet, especially with typically rich oxygen vacancies in γ-WO_3_ [50,54,55], rising the sensor response. A great number of W atoms are present on the (020) crystal plane, indicating the presence of a great number of dangling bonds, favorable for the sensor performance. The third crystal plane (200) consists of a mixture of O and W atoms and the number of W atoms is less than that on the (020) facet [53]. Possessing both advantages of easy oxygen chemisorption and high reactivity at the dangling bonds of W atoms, the (200) facet shows reasonable sensing response to methane at a relatively low optimum operating temperature of 90 °C.

**Figure 9:**
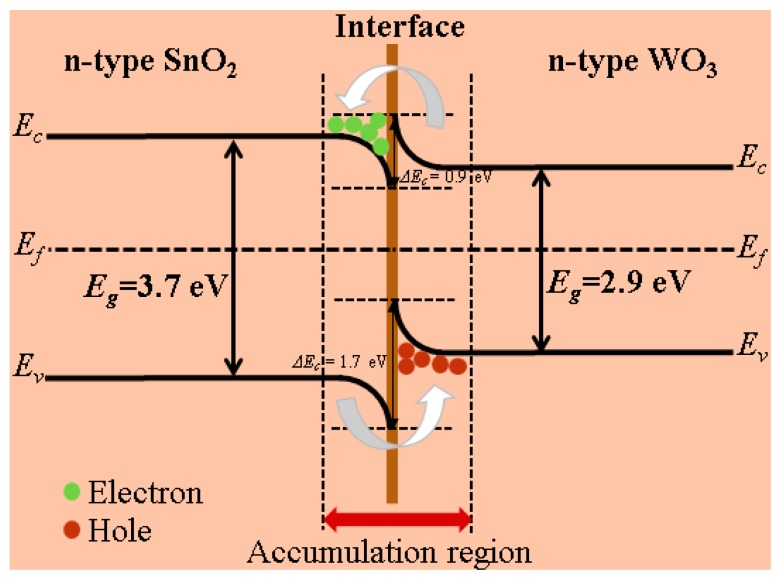
Energy band diagram of the WO_3_-SnO_2_ (n–n) heterojunction at thermal equilibrium.

Both of the WO_3_ nanosheets and the SnO_2_ nanoparticles were lightly n-doped. Thus, a n–n heterojunction formed at the interface. The band gap *E*_g_ was 2.9 eV and 3.7 eV and the electron affinity *χ* was −3.3 eV and −4.2 eV for WO_3_ and SnO_2_, respectively [56,57,58], resulting in Δ*E*_c_ = −(*X*_Sn_ − *X*_W_) = 0.9 eV, Δ*E*_v_ = −(*X*_Sn_ + *E*_g,Sn_ − *X*_W_ − *E*_g,w_) = 1.7 eV. The Fermi levels were located slightly above the middle level of the band gap. Band bending occurred at the n–n heterojunction trapping electrons and holes near the interface in SnO_2_ and WO_3_, respectively, at thermal equilibrium (Figure 9). Subsequently, the higher electron density in the conduction band of SnO_2_ at the interface enhanced the local oxygen chemisorption. Otherwise, the lower electron density in WO3 reduced the local chemisorption deteriorating the sensing performance. However, the N_2_ adsorption–desorption investigation revealed smaller pore sizes and a larger specific surface area of the SnO_2_ nanoparticles as compared to the WO_3_ nanosheets as discussed above, which made SnO_2_ the dominating phase for oxygen adsorption. Therefore, such a heterojunction improved the performance of the gas sensor. Surely, the much-increased specific surface area by SnO_2_ loading enhanced the sensor sensitivity by itself.

The defects formed at the heterojunction could be an additional factor contributing to a better gas-sensing performance. A large lattice mismatch between WO_3_ and SnO_2_ as observed from the TEM investigation and even the different crystal structures of the monoclinic WO_3_ and the rutile SnO_2_ resulted in high defect density at the interface. The defects with dangling bonds served as adsorption and highly reactive sites enhancing the sensor sensitivity and a low optimum operating temperature of 90 °C.

## 4. Conclusions

In summary, SnO_2_-loaded WO_3_ nanosheets were prepared by a simple impregnation method and a subsequent calcination treatment using SnO_2_ and WO_3·_H_2_O obtained by the hydrothermal method as precursors. The synthesized pure and SnO_2_-loaded WO_3_ nanosheets had good crystallinity and high purity proved by a series of characterization methods, and SnO_2_ nanoparticles with a particle size of about 2 nm were uniformly dispersed on the surface of WO_3_ nanosheets. Studies of gas-sensing performance showed that the load of SnO_2_ nanoparticles enhanced the sensor sensitivity by a maximum factor of 1.4 as compared to the pure WO_3_ nanosheets. Satisfactory repeatability and long-term stability were ensured. The gas-sensing mechanisms were discussed as follows: The observed dominant exposed (200) facet of the WO_3_ nanosheets, possessing a good balance between easy oxygen chemisorption and high reactivity at the dangling bonds of W atoms, had a reasonable response value (~1.5) at a low optimum operating temperature (90 °C) without adding any catalyst. Moreover, the electron accumulation layer in SnO_2_ enhanced the oxygen adsorption at the surface with SnO_2_ as the dominant phase for oxygen adsorption revealed by N_2_ adsorption–desorption. The dramatically increased specific surface area from 10.5 m^2^/g to 57.7 m^2^/g contributed to the improvement of sensor sensitivity as well. Finally, defects formed at the heterojunction were discussed as adsorption and highly reactive sites favorable for gas-sensing performance.
